# Storage Impact on the Physicochemical and Microbiological Stability of Apricot, Cherry, Raspberry, and Strawberry Jams

**DOI:** 10.3390/foods14101695

**Published:** 2025-05-11

**Authors:** Ancuta Elena Prisacaru, Cristina Ghinea, Eufrozina Albu, Sergiu Pădureţ

**Affiliations:** Faculty of Food Engineering, Stefan cel Mare University of Suceava, 720229 Suceava, Romania; ancuta.prisacaru@fia.usv.ro (A.E.P.); e.albu@fia.usv.ro (E.A.); sergiu.paduret@fia.usv.ro (S.P.)

**Keywords:** fruit jams, physicochemical parameters, storage temperature, yeast and molds

## Abstract

Fruits, such as apricots, cherries, raspberries, and strawberries, are very often processed into jams to extend their shelf life. Jams are highly appreciated by consumers, and their storage conditions are important for preserving their quality. This study investigates the impact of storage on the microbiological quality and physicochemical parameters of five commercial fruit (apricot, sour cherry, white cherry, raspberry, and strawberry) jams. The pH, titratable acidity, moisture, sugar content, viscosity, and color were evaluated immediately after opening the jam containers and during storage at 2–4 °C and 20 °C. The total number of mesophilic aerobic germs (TMAG) and the total number of yeasts and molds (TYM) were also determined. The samples were analyzed during storage at 14, 42, and 56 days after opening the jam jars. The pH of the fruit jam samples varied from 2.91 (strawberry jam) to 3.47 (sour cherry jam) and decreased during storage, while the titratable acidity (TA) ranged from 0.46 (cherry and raspberry jams) to 0.52% malic acid (apricot jam) and increased during storage, regardless of the storage temperature. The moisture content was between 15.78% (apricot jam) and 21.82% (raspberry jam), and decreased in all the jam samples during storage. The sugar content (30.40–44.59 g per 100 g of jam) was typical for low-sweetened jams and increased during storage. Also, the viscosity of the fruit jam samples increased during storage (more in the samples stored at room temperature). Under the storage conditions, all the jam samples lost their specific color. Immediately after opening the jam jars, no yeasts or molds were found in the apricot and sour cherry jam samples. The highest number of yeasts and molds was detected in the white cherry jam samples (4.25 log_10_ CFU/g). The TYM increased during storage, as did the TMAG. The time–temperature interaction factor influenced the physicochemical and microbiological properties of the jam samples.

## 1. Introduction

Worldwide, fruits are widely consumed raw and processed for their pleasant aromas and tastes [1,2]. At the global level, over 3.7 million tons of apricots (*Prunus armeniaca*), 940,000 tons of raspberries (*Rubus idaeus*), 10 million tons of strawberries (*Fragaria ananassa*), 1.5 million tons of sour cherries (*Prunus cerasus*), and 2.9 million tons of cherries (*Prunus avium*) were produced in 2023 [3]. The countries which produced the largest quantities of these fruits were Turkey for apricots and cherries, the Russian Federation for raspberries and sour cherries, and China for strawberries [4]. In the European Union, the leading countries in 2023 for the production of apricots, raspberries, strawberries, cherries, and sour cherries were as follows: Italy (with 207,190 tons of apricots), Poland (with 96,100 tons of raspberries and 168,700 tons of sour cherries), Spain (with 329,280 tons of strawberries), and Greece (with 113,580 tons of cherries) [4]. In the same year, Romania produced 36,560 tons of cherries and 29,000 tons of sour cherries, 24,550 tons of apricots, 20,640 tons of strawberries, and 430 tons of raspberries [3]. Many fruits have a very short storage life due to their perishable nature and are often processed into jams, jellies, purees, and juices [2,5]. Italy, with 150,758 tons, Chile, with 135,482 tons, and India, with 127,959 tons, were the main exporters of jams, fruit jellies, marmalades, fruit or nut purées, and fruit or nut pastes in 2023 [6]. Turkey is the main exporter of apricots, cherries, raspberries, and strawberry jams, according to [7]. France, Germany, and Spain are the countries with the largest volume of jam consumption in the European Union [8]. Jams are popular due to their year-round availability, low cost, and organoleptic properties, being a good source of energy and carbohydrates (66–68%) [9]. Usually, fruits, sugar, and edible acids are used to make jams, so, by increasing the soluble solids content, stable and preserved foods are obtained [10]. According to Hashim et al. [11], a good jam has a soft, uniform consistency with no visible pieces of fruit, a vivid color, a good fruity flavor, and a jelly-like jam texture that is easy to spread but has no free liquid. Fruits like apricots, sour and white cherries, strawberries, and raspberries are widely used for jam manufacturing. Apricots contain 79.86–88.60% water, 0.66–1.33% protein, 0.10–0.57% fat, and 7.28–13.30% carbohydrates [12]. Alajil et al. [13] investigated six apricot genotypes and determined a total soluble solids (TSS) content of between 12.13 and 17.82 Brix, a titratable acidity between 1.88 and 2.53%, while the total sugar content ranged from 9.79 to 15.59 g/100 g, and the sweetness index ranged from 13.58 to 22.30. Apricots contain citric acid (55% of the organic acids present in apricots); malic acid (25% of the organic acids), responsible for fruit sourness; and succinic acid (20% of the organic acids). Also, apricots have a high carotenoids content (0.44–3.50 mg/100 g), and a total phenolic content from 25.31 to 89.95 mg GAE/100 g [13]. Sour cherries contain 81–88% water, <1.0% protein and fat, and 10.1–15.1% carbohydrates [14,15,16], while sweet cherries contain 80–83% water, 0.8–1.4% protein, 0.2–0.7% fat, and 12.2–17.0% carbohydrates [17]. Sour cherries are high in vitamin A (1283 IU/100 g) and total phenolics (254.1 mg GAE/100 g FW) according to Blando and Oomah [18]. Sokół-Łętowska et al. [19] determined a total organic acid content between 1294.4 mg/100 g FW and 2300.5 mg/100 g FW in sour cherries. The organic acids identified in sour cherries were malic (78.2–88.3% of the total organic acids), malonic (11.2–21.3%), and oxalic, shikimic, and fumaric (in lower concentrations) [19]. The fruit’s color depends on the anthocyanin content of the sour cherries, which can vary between 17.97 and 131.28 mg/100 g FW depending on the variety, while sweet cherries can have 1.2 to 900.0 mg/100 g FW [19]. The total phenolic content of sour cherries can vary between 96.56 and 268.98 mg/100 g FW depending also on the variety, maturity, agronomic factors, and climatic conditions [19]. White cherries (*Prunus avium* ‘Rainier’) have an anthocyanin total concentration of 6.4 mg 100 g^−1^ FW, a carotenoid concentration of 8 µg g^−1^ FW, a total chlorophyll concentration of 5.4 µg g^−1^ FW, and a total phenolic content of 108 mg chlorogenic acid equivalents 100 g^−1^ FW, according to Reyes-Manríquez et al. [20]. Strawberries contain 86.3–90.9% water, 0.57–0.73% protein, 0.13–0.15% fat, and 7.68–12.4% carbohydrates [21,22]. The total phenolic content of strawberries can vary from 607 to 1314 mg GAE/100 g [22], and the total anthocyanin content from 150 to 800 mg/kg of the fresh weight [21]. Vitamin C, responsible for the antioxidant capacity of strawberries, can range from 13.84 to 71.20 mg ascorbic acid/100 g FW [23]. Urün et al. [24] found that the total phenolic content of strawberries ranges from 99.02 to 158.37 mg GAE 100 g^−1^ FW. Oxalic, L-ascorbic, citric, malic, fumaric, and succinic are the main organic acids found in strawberry fruits. Raspberries contain 81.06–87.04% water, 6.78–9.16% protein, and 3.21–5.88% fat [25]. Akimov et al. [26] reported the citric acid content in raspberries to be between 466 and 1750 mg/100 g, the level of ascorbic acid between 13.6 and 31.1 mg/100 g, and high anthocyanins content (62.4–83.6 mg/100 g fresh fruit). Yu et al. [27] found that citric acid (2.95–13.85 g/100 g DW) is the main organic acid in raspberry fruits, followed by malic acid (0.31–2.37 g/100 g DW). The total phenolic content of raspberries can vary between 0.77 and 1.19 g/100 g DW according to Yu et al. [27]. Therefore, fruits are essential sources of vital nutrients in the diet, such as carbohydrates, vitamins, bioactive compounds, and minerals. Over 30% of these fruits spoil, and fruit processing is one of the ways to reduce post-harvest losses [28]. Therefore, to avoid post-harvest losses and to increase shelf life, fruit should be processed into shelf-stable products, such as juices, jams, and jellies. The production of fruit jams includes steps such as extraction, clarification, boiling, and stabilization. However, the level of sweetness depends very much on the concentration of each ingredient and the processing parameters [29]. The ideal fruits for obtaining jams have three factors in common: a high pectin content, a relatively low pH, and a high content of total soluble substances. Chauhan et al. [30] reported 0.32% pectin for apricot pulp, Lara-Espinoza et al. [31] indicated a content of 0.4% pectin in cherries, Stanisavljevic et al. [32] obtained a pectin content between 0.39 and 0.45% in raspberries, while Novikova et al. [33] reported a pectin content between 0.26 and 0.77% in strawberries. Muñoz-Almagro et al. [34] determined a pH of 3.4 for strawberries and 3.1 for raspberries, Chauhan et al. [30] reported a pH of 3.37 for apricot pulp, while Silva et al. [35] obtained a pH between 3.70 and 3.94 for cherries. A total soluble solids (TSS) content of 15.20 °Brix for apricot pulp was obtained by Chauhan et al. [30], Silva et al. [35] indicated a TSS content between 11 and 25 °Brix for cherries, the TSS content of strawberries can be between 7.16 and 9.65 °Brix according to Patel et al. [36], while for raspberries it can range between 9 and 11 °Brix [37]. Jams can be stored for extremely long periods. The influence of the storage time on the quality of different jams has been investigated in different studies: Touati et al. [2] monitored the total soluble solids, titratable acidity, color, free amino acids, total sugars, and sensory attributes of apricot jam (purchased from Algerian markets) stored at 5 °C, 25 °C, and 37 °C for 60 days; Pavlova et al. [38] evaluated the physicochemical parameters, such as soluble solids, sugars, total acids, pH, vitamin C, fat, protein, and microbiological safety, of raspberry and peach jams after 15 and 90 days of storage; and Poiana et al. [39] evaluated the changes in vitamin C, total phenolic content, total anthocyanins, and color parameters of strawberry, sweet cherry, and cherry jams during storage at 20 °C after 1 and 3 months. The storage conditions are important factors for the quality of jams. This study aimed to analyze how consumer storage conditions influence the physicochemical and microbiological properties of fruit jams over time. Most jam manufacturers state on the label to “store the product in clean, well-ventilated areas, free from frost and sunlight”, and recommend a storage temperature between 5 and 25 °C. Some of them also mention that, after opening, the jam should be kept in the refrigerator and consumed within 30 days. On this basis, an innovative aspect of this research is that it provides useful information to both producers and consumers by monitoring the physicochemical and microbiological changes in different commercial fruit jams during 56 days (8 weeks) of storage. Therefore, the objective of this study was to monitor the physicochemical and microbiological stability of jams obtained from five fruit varieties (strawberries, sour cherries, apricots, white cherries, and raspberries) during their storage at refrigeration temperature (2–4 °C) and room temperature (20 ± 2 °C). Apricots, strawberries, raspberries, sour cherries, and white cherries are not available for fresh consumption all year round, but the jams made from these fruits are much more available and are also highly appreciated by consumers; therefore, they were selected for this study. The pH, titratable acidity, moisture, sugar content, viscosity, and color were determined. The total number of yeasts and molds and the total number of mesophilic aerobic germs were also determined.

## 2. Materials and Methods

### 2.1. Samples

**Figure 1:**
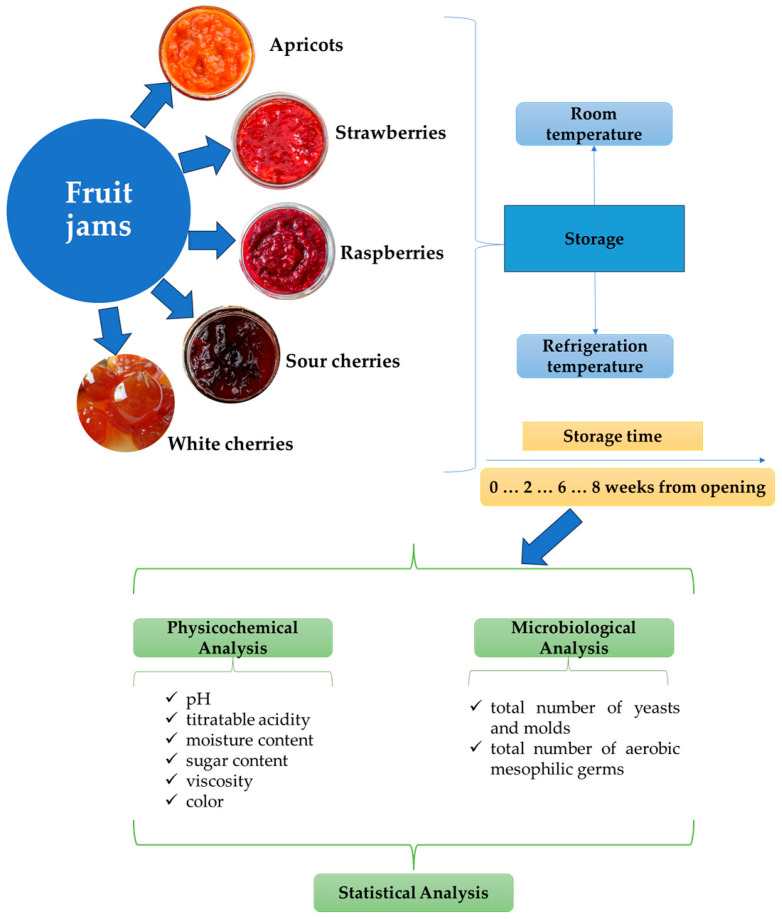
Experimental plan of this study.

Five varieties of fruit jams were purchased from supermarkets in Suceava, Romania. The samples consisted of apricot (AJ), raspberry (RJ), strawberry (SJ), sour cherry (SCJ), and white cherry (WCJ) jams. The fruit jams were produced in 2022 by Romanian producers and were packaged in 370 g jars. The nutritional and compositional properties of jams are presented in Table 1. Products were purchased before the expiration date and transported to the university laboratory. After opening, the jars with jam were stored at room temperature (20 ± 2 °C) and refrigeration temperature (2 ± 4 °C) in order to evaluate the influence of temperature and storage period on their properties. The jars were not placed in a sterile environment because we intended to investigate them under storage conditions similar to those of the average consumer. Laboratory tests were conducted in the fall of 2022, over a period of eight weeks (56 days). The experimental plan is illustrated in Figure 1.

### 2.2. Chemicals, Working Standard Solutions, and Culture Media

All chemicals and reagents utilized in the physicochemical experiments of this study, including sodium hydroxide (NaOH), phenolphthalein, lead (Pb) acetate solution, 8% sodium phosphate, 20% potassium iodide, 26.5% sulfuric acid (H₂SO₄), 1% amylum, 0.1 N sodium thiosulfate, 30% hydrochloric acid (HCl), 45% sodium hydroxide (NaOH), phenolphthalein, sodium carbonate (Na₂CO₃), and copper(II) sulfate pentahydrate (CuSO₄·5H₂O) were procured from Sigma-Aldrich (Sigma-Aldrich Chemie GmbH, Taufkirchen, Germany). All reagents were of analytical grade. Nutrient medium Dichloran Rose Bengal Chloramphenicol Agar and Plate Count Agar were purchased from Merck, Darmstadt, Germany.

### 2.3. Physicochemical Analysis

*pH determination*: pH of fruit jams was measured by using a digital pH meter (Mettler Toledo), which was previously calibrated. The pH was determined by inserting the pH electrode directly into a container of fruit jam sample [40,41]. The pH meter was rinsed with deionized water and dried immediately after each reading. The pH reading for each fruit jam sample was performed three times for each sample.

*Determination of titratable acidity of fruit jam samples*: Titratable acidity was determined by the acid–base method: 5 g of jam sample was homogenized with 50 mL of distilled water and the mixture was titrated with NaOH in the presence of phenolphthalein until faint pink [40,42]. Titratable acidity was expressed as % malic acid.

*Determination of moisture content of fruit jam samples*: Approximately 5 g of well-homogenized sample was accurately weighed into a pre-dried container (approximately 75 mm wide and 25 mm deep). The vessel was placed in an oven at 105 ± 2 °C until constant mass was obtained from two successive weighings [40].

*Determination of sugar content in fruit jam samples*: The sugar content of fruit jam samples was determined by applying the Luff–Schoorl method [43,44,45]. Approximately 5–10 g of jam was weighed, diluted with distilled water in a 100 mL volumetric flask, and homogenized. Pb acetate was added dropwise until clarification occurred. The mixture was diluted to volume, filtered, and 50 mL of the filtrate was transferred to a 200 mL volumetric flask. After the addition of 8% sodium phosphate to remove turbidity, the solution was diluted, shaken, and filtered. A 25 mL aliquot of the Pb-free filtrate was mixed with 25 mL of Luff–Schoorl solution and 10 mL of 30% HCl in a 100 mL volumetric flask. The solution was heated at 60–70 °C for 10 min, cooled, and neutralized with 45% NaOH in the presence of phenolphthalein. After further dilution, a 25 mL portion was refluxed with 25 mL of Luff–Schoorl solution and boiling stones for 10 min, then rapidly cooled. The mixture was acidified with 26.5% H₂SO₄, treated with 15 mL of 20% KI, and titrated with sodium thiosulfate until yellow. After adding 1% amylum, titration continued until the blue color disappeared. A blank titration was performed using distilled water.

*Viscosity determination*: The viscosity of the samples was determined at 25 °C using a Brookfield viscometer, equipped with a T-shaft spindle, operating at a rotational speed of 20 rpm. The measurement was conducted following the standard procedures outlined by the AOAC [46], ensuring consistency and reliability in viscosity assessment.

*Determination of fruit jams’ colors*: Color analysis of the jam samples was performed using the CIELab system with a Konica Minolta CR-400 ChromaMeter (Konica Minolta, Tokyo, Japan). This method characterizes color through the L* (lightness), a* (red–green), and b* (yellow–blue) coordinates. Samples were spread evenly in transparent Petri dishes and placed on a white surface for measurement. The total color difference (∆E) was calculated to assess variations between samples [47].

### 2.4. Microbiological Analysis

The microbiological load was analyzed for each fruit jam sample, namely total number of mesophilic aerobic germs (TAMG) and total number of yeasts and molds. The fruit jam samples were processed under sterile conditions to obtain an extract by processing 10 g of product with 90 mL of sterile solvent. From the initial dilution (sample extract), a series of decimal dilutions were made on the culture media for the counting and identification of micro-organisms. Standard ISO 21527-2/2008 [48] was considered for the determination of total number of yeasts and molds. The number of yeasts and molds was established by cultivation on Dichloran Rose Bengal Chloramphenicol Agar culture medium purchased from Merck, Darmstadt, Germany. From the dilutions established by the product standard, 1 cm^3^ was inoculated into Dichloran Rose Bengal Chloramphenicol Agar culture medium. The plates were incubated at 25 ± 1 °C for five days. Visible colonies of typical molds were counted and reported per gram of sample [48].

Standard SR EN ISO 4833-2/2014 [49] was considered for the determination of the TAMG. The total number of mesophilic aerobic germs (TAMG) was determined by cultivation on Plate Count Agar culture medium (PCA), purchased from Merck, Darmstadt, Germany. From the dilutions established by the product standard, 0.1 cm^3^ was spread on the surface of the Plate Count Agar culture medium. The plates were incubated at 30 ± 2 °C for 72 ± 3 h. The number of micro-organisms per gram of the test sample was calculated from the number of colonies obtained from the plates containing fewer than 300 colonies [49]. Funke Gerber ColonyStar (Funke Gerber, Berlin, Germany) was used for colony counting.

### 2.5. Statistical Analysis

The physicochemical and microbiological results were statistically evaluated with Minitab version 17 (State College, PA, USA). ANOVA (95% confidence interval (*p* < 0.05)) with Tukey’s test, principal component analysis (PCA), and Pearson’s correlation were conducted.

## 3. Results and Discussion

### 3.1. Storage Effects on the pH, Titratable Acidity, and Moisture Content of Fruit Jam Samples

**Figure 2:**
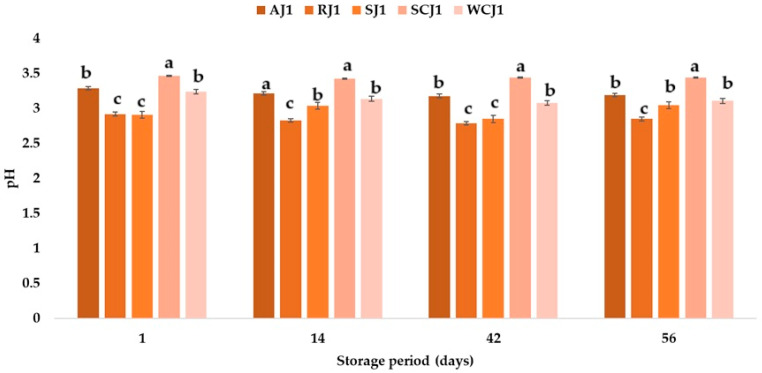
pH of fruit jam samples during storage at room temperature. Means with different lowercase letters (a–c) indicate significant differences (*p* < 0.05) between samples.

**Figure 3:**
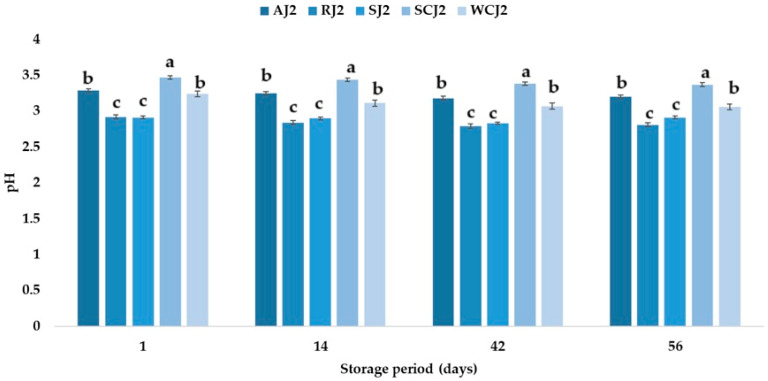
pH of fruit jam samples during storage at refrigeration temperature. Means with different lowercase letters (a–c) indicate significant differences (*p* < 0.05) between samples.

Figure 2 and Figure 3 show the pH variations in the jam samples stored at room and refrigeration temperatures, respectively. At the moment of opening the jam containers, the pH of the samples varied between 2.91 (strawberry jam) and 3.47 (sour cherry jam). Khan et al. [50] determined an initial pH value of 3.20 for strawberry jam, and Picariello et al. [51] obtained a pH of 3.28 and 3.57 for sour cherry and white cherry jams, respectively. Martinsen et al. [52] reported pH values between 3.20 and 3.26 for strawberry jams and between 2.98 and 3.04 for raspberry jams. These values are higher than those obtained in the present study for the strawberry (2.91) and raspberry (2.92) jams. Touati et al. [2] reported a value of 3.54 for apricot jam, Aslanova et al. [53] obtained a pH of 3.34, while the pH of the apricot jam in the present study was 3.29. Mahdi [54] obtained the following pH values for local commercial jams—3.74–3.98 for strawberry jam and 3.96–4.09 for apricot jam—while for imported jams they reported a pH between 2.93 and 3.91 for strawberry jam and between 2.09 and 4.15 for apricot jam. A pH less than 4.0 indicates that jams are microbiologically stable according to Uribe-Wandurraga et al. [55]. The pH of fruit jams is usually between 2.8 and 3.5, as the proper gelation of pectin only occurs in this range [56], while, according to Damiani et al. [57], a jam should have a pH between 3.0 and 4.0. The pH values of the jam samples decreased after 14 days of storage at room temperature by 1.15% for the sour cherry jam sample, by 2.12% for the apricot jam sample, by 3.08% for the raspberry jam sample, by 3.09% for the white cherry jam sample and increased by 4.46% for the strawberry jam sample. For all the jam samples kept at refrigeration temperature, the pH values decreased after 14 days of storage; the highest decrease was recorded for the white cherry jam sample (4.01%) and the lowest for the strawberry jam sample (0.34%). After 42 days of storage, the pH values of the jam samples maintained at both refrigeration and room temperature decreased further. After 56 days of storage at room temperature, it was observed that the highest pH value was for the sour cherry jam sample (3.44), followed by the apricot, white cherry, and strawberry jam samples (the pH values for these three samples did not have statistically significant differences), while for the raspberry jam sample, the pH value was 2.85 (Figure 1).

As regards the pH of the jam samples kept for 56 days at refrigeration temperature, it was observed that the sour cherry jam sample had the highest pH of all the samples analyzed (3.37), followed by the apricot jam and white cherry jam, at 3.20 and 3.06, respectively (Figure 2).

**Figure 4:**
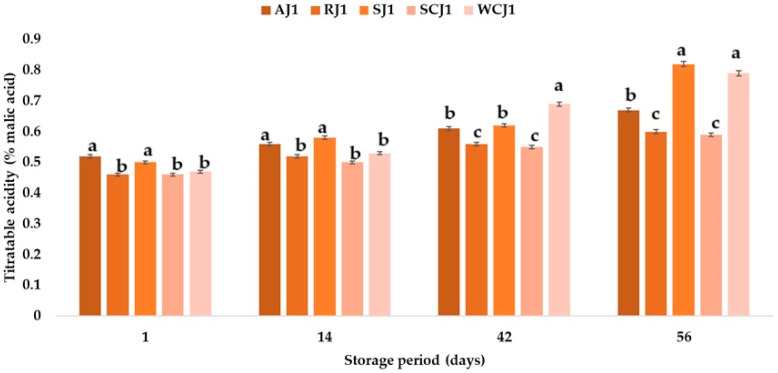
Titratable acidity (% malic acid) of fruit jam samples during storage at room temperature. Means with different lowercase letters (a–c) indicate significant differences (*p* < 0.05) between samples.

**Figure 5:**
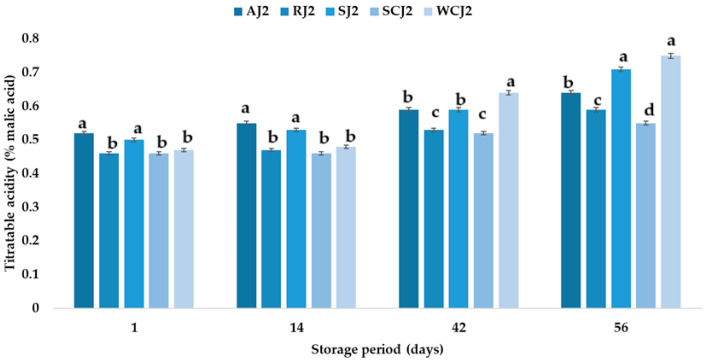
Titratable acidity (% malic acid) of fruit jam samples during storage at refrigeration temperature. Means with different lowercase letters (a–d) indicate significant differences (*p* < 0.05) between samples.

The pH values of the strawberry and raspberry jam samples were 2.91 and 2.81. The ranking of the jam samples according to their pH (in descending order of values) after storage at both room temperature and refrigeration temperature is SCJ > AJ > WCJ > SJ > RJ. A decrease in the pH of apricot jam during storage was also observed by Touati et al. [2], who indicated that the time–temperature interaction factor had a significant effect on this parameter. Khan et al. [50] also reported a decrease in the pH values of strawberry jam during storage, from 3.20 to 2.91. One of the physicochemical parameters that affects the quality of a product is the titratable acidity. To a large extent, acidity protects against the development of micro-organisms [2]. The titratable acidity ranged from 0.46 (cherry and raspberry jams) to 0.52% malic acid (apricot jam). The strawberry jam had a titratable acidity of 0.50% malic acid, while the white cherry jam had 0.47% malic acid. In [58], a maximum of 0.5% malic acid was stipulated for products preserved with sugar. Makanjuola and Alokun [59] determined a titratable acidity of 0.68% malic acid for commercial strawberry jam, while Uzun-Viruzab et al. [60] reported between 0.31 and 0.48% malic acid in different jam samples. The changes in the titratable acidity (% malic acid) of the fruit jam samples during storage at room temperature and refrigeration temperature are illustrated in Figure 4 and Figure 5. The titratable acidity increased during the storage period regardless of the temperature at which the samples were kept. The same was observed by Touati et al. [2]; Khan et al. [50] also reported that the acidity of strawberry jam increased from 0.68 to 0.86% during storage. In the present study, after 14 days of storage at room temperature, the titratable acidity of the strawberry jam sample increased the most, by 16%, compared to the initial acidity, and the titratable acidity of the apricot sample increased only by 7.69%. For the other jam samples, increases in the titratable acidity values of 8.70% (sour cherry jam), 12.77% (white cherry jam), and 13.04% (raspberry jam) were recorded. The increase in the titratable acidity values continued after 42 days of storage at room temperature, but the highest increase was recorded for the white cherry jam sample, followed by the strawberry jam sample (increases of 46.81% and 24.00% compared to the initial values). At the end of the storage period (after 56 days), the strawberry and white cherry jam samples had the highest values for titratable acidity (0.82 and 0.79% malic acid), while the sour cherry jam and raspberry jam samples had the lowest values (0.59 and 0.60% malic acid). The titratable acidity values increased over time also in the jam samples stored at refrigeration temperature, but the increase was lower after 14 days (values between 2.13 and 6%) than in the samples stored at room temperature (with increases between 7.69 and 16%). Thus, the largest increase was recorded for the strawberry jam sample (6%), while for the sour cherry jam sample no change in titratable acidity was observed. After 42 days of storage at refrigeration temperature, it was observed that the titratable acidity values increased the most for the white cherry jam sample (36.17%) and the strawberry jam sample (18%), and the smallest increases were determined for the raspberry and sour cherry jam samples. At the end of the 56 days of storage, it was observed that the titratable acidity of the white cherry jam samples increased by 59.57% compared to the initial value (immediately after opening), followed by that of the strawberry jam samples (42%), while for the sour cherry jam samples, the titratable acidity increased only by 19.57% (being the smallest increase). The ranking of the jam samples according to their titratable acidity after storage for 56 days at room temperature is SJ > WCJ > AJ > RJ > SCJ, and after storage at refrigeration temperature is WCJ > SJ > AJ > RJ > SCJ. According to Shahid et al. [61], the increase in acidity during storage can be attributed to the oxidation of reducing sugars; the degradation of polysaccharides, pectin substances, and uronic acid; or the addition of chemical additives.

During jam storage, the pH decreases due to increased acidity, which leads to pectin breakdown during storage. The pH values influence microbial growth. Yeasts and molds can tolerate lower pH conditions than most bacteria, but very few foods have pH values low enough to completely inhibit the growth of micro-organisms. The observed decline in the pH values during storage—from an initial maximum of 3.47 in the sour cherry jam to progressively lower values—combined with the concomitant increase in titratable acidity (reaching up to 0.52% malic acid in apricot jam), reflects a shift toward a greater acidity in all the jam samples over time. These changes are indicative of ongoing acidification, which can arise from microbial metabolic byproducts or from physicochemical reactions, such as the breakdown of sugars and organic acids. This acidification process has dual implications: while it may enhance microbial suppression in some cases, it can also adversely affect the organoleptic properties, particularly flavor, rendering the product less palatable over time. Moreover, this phenomenon underscores the importance of monitoring the pH and titratable acidity as critical indicators of both microbial stability and sensory quality during storage.

**Figure 6:**
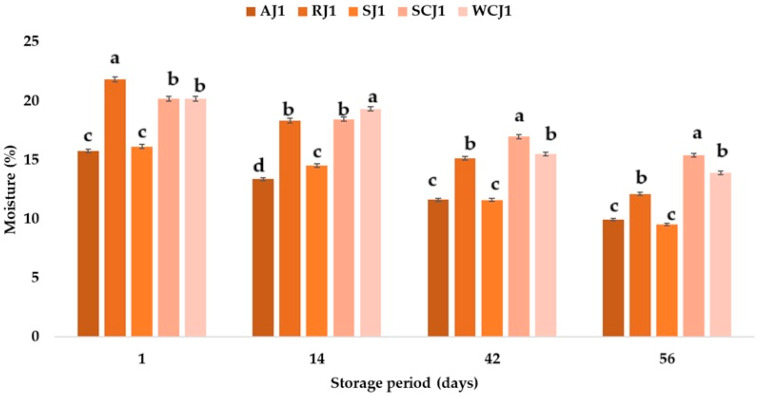
Moisture content (%) of fruit jam samples during storage at room temperature. Means with different lowercase letters (a–d) indicate significant differences (*p* < 0.05) between samples.

**Figure 7:**
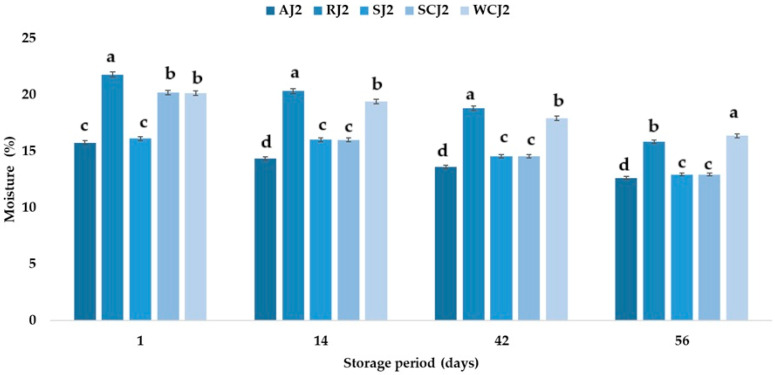
Moisture content (%) of fruit jam samples during storage at refrigeration temperature. Means with different lowercase letters (a–d) indicate significant differences (*p* < 0.05) between samples.

The shelf life of a product depends on its water content, so the higher it is, the shorter the shelf life. The differences in the water content of the samples depended on the raw materials used in the manufacture of the jam assortments. The moisture content of the jam samples ranged between 15.78% (apricot jam) and 21.82% (raspberry jam), and decreased in all the jam samples during storage at room temperature (Figure 6). According to Naeem et al. [9], the moisture content of apricot jam can vary between 27.91 and 42.01%, while for strawberry jam it can vary between 24.79 and 43.22%. Mahdi [54] reported the moisture content of commercial local strawberry jams to be between 31.55 and 33.08%, while for apricot jam, they determined the moisture content to be between 32.77 and 36.86%. Kántor et al. [62] obtained moisture contents of 59.0% for apricot jam, 57.7% for cherry jam, 55.3% for strawberry jam, and 58.6% for raspberry jam. These values are higher than those obtained in the present study. According to Naeem et al. [9], the long shelf life of jams can be indicated by their low moisture content. After 14 days of storage, it was observed that the moisture content of the white cherry jam samples decreased the least (by 4.16%), while the moisture values of the raspberry and apricot jam samples decreased the most (15–16%). After 42 days of storage at room temperature, the moisture content of the jam samples decreased the most in the raspberry and strawberry jam samples (30.52% and 28.07%), compared to the moisture values of the samples immediately after opening. For the same jam samples (raspberry and strawberry), the largest decreases in moisture values (44.41% and 40.89%) were recorded 56 days after storage. In the case of the samples stored at refrigeration temperature (Figure 7), it was observed that the moisture content of the jam samples also decreased during storage as follows: after 14 days of storage, the highest decrease in moisture was in the sour cherry jam sample (20.69%), and the lowest in the strawberry (0.62%); after 42 days, the same samples had the highest and lowest percentage decreases in moisture (27.87% and 9.73%, respectively, compared to the moisture values obtained immediately after opening the samples); and after 56 days of storage, a decrease in moisture content of 35.94% was determined for the sour cherry jam sample, while the lowest decrease in moisture content was obtained for the white cherry jam sample (18.79%). The ranking of the jam samples after storage for 56 days at room temperature according to their moisture content is SCJ > WCJ > RJ > AJ > SJ, and after storage at refrigeration temperature is WCJ > RJ > SJ > SCJ > AJ.

The moisture content influences the shelf life of food products. Fluctuations in the moisture content may be related to the activity of micro-organisms and enzymes. The sugar content can also influence the moisture content of a food.

A consistent decrease in the moisture content—most notably of 21.82% in the raspberry jam—and an increase in the sugar concentration (up to 44.59 g per 100 g of product) were noted during storage. These trends suggest a concentration effect, likely due to water evaporation, which not only alters the products’ texture and perceived sweetness, but also significantly affects their water activity. Lower water activity may reduce the risk of microbial proliferation; however, in low-sugar formulations, such as those studied here, the protective osmotic effect is less pronounced. The interplay between reduced moisture and elevated sugar content thus carries direct implications for both microbiological safety and consumer sensory experience, particularly in terms of the sweetness balance and mouthfeel.

### 3.2. Storage Effect on Sugar Content of Fruit Jam Samples

**Figure 8:**
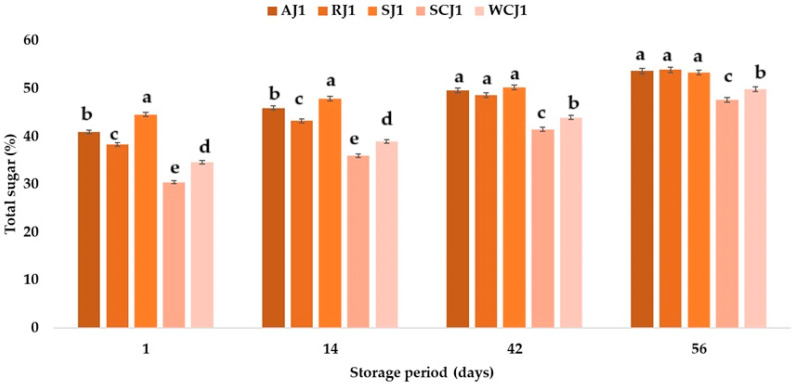
Total sugar (%) of fruit jam samples during storage at room temperature. Means with different lowercase letters (a–e) indicate significant differences (*p* < 0.05) between samples.

**Figure 9:**
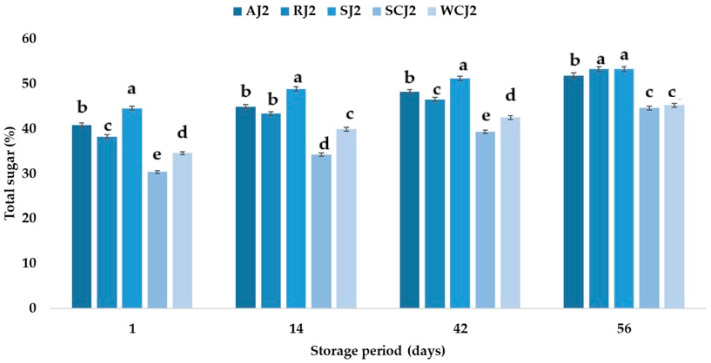
Total sugar (%) of fruit jam samples during storage at refrigeration temperature. Means with different lowercase letters (a–e) indicate significant differences (*p* < 0.05) between samples.

The strawberry jam sample had the highest sugar content after opening the jars (44.59%), followed by the apricot jam (40.92%), raspberry jam (38.32%), white cherry jam (34.63%), and sour jam (30.40%). Jribi et al. [63] obtained 25–51 g sugar per 100 g of strawberry jam, which is typical for low-sweetened jams, while Touati et al. [2] determined 64.88 g total sugar per 100 g of apricot jam, and Vilela et al. [64] reported 70.70 g total sugar per 100 g of strawberry jam, 66.80 g total sugar per 100 g of cherry jam, and 60.20 g total sugar per 100 g of raspberry jam. The results obtained in this study show that the total sugar content of the jam samples increased during storage at room temperature (Figure 8). The highest increase in the total sugar content was obtained for the sour cherry jam sample (which increased by 18.29% after 14 days of storage, by 36.41% after 42 days of storage, and by 56.71% after 56 days of storage), and the lowest increase was observed in the case of the strawberry jam (which increased by 7.47% after 14 days of storage, by 12.69% after 42 days of storage, and by 19.62% after 56 days of storage). At the end of the storage period, the results show that the total sugar content of the raspberry, apricot, and strawberry jam samples were not statistically significantly different (Figure 8). The increase in the sugar content was lower in the case of the samples stored at room temperature, but the highest and lowest increases in sugar content were also recorded for the sour cherry jam and strawberry jam samples, regardless of the storage duration (Figure 9). The increase in the sugar content of jams during storage can be associated with the decrease in moisture during storage [65]. Touati et al. [2] reported a decrease in the sugar content of apricot jam during storage at 5 °C, 25 °C, and 37 °C. The decrease in the sugar content was lower in the case of the samples maintained at lower temperatures. The decrease in sugar content could be due to the contribution of the reducing sugars to non-enzymatic browning and hydroxymethylfurfural formation [38]. From the present study, based on the obtained results regarding the sugar content, it can be concluded that the raspberry, strawberry, and white cherry jam samples stored better for 14 days at room temperature (the sugar content changed less in these samples compared to that obtained for the samples stored at refrigeration temperatures), but for up to 42 days of storage, it was only in the strawberry jam sample stored at room temperature that the sugar content changed less. The ranking of the jam samples after storage for 56 days at room temperature according to their total sugar content is RJ > AJ > SJ > WCJ > SCJ, and after storage at refrigeration temperature is RJ > SJ > AJ > WCJ > SCJ.

### 3.3. Viscosity Changes During Storage of Fruit Jam Samples

**Figure 10:**
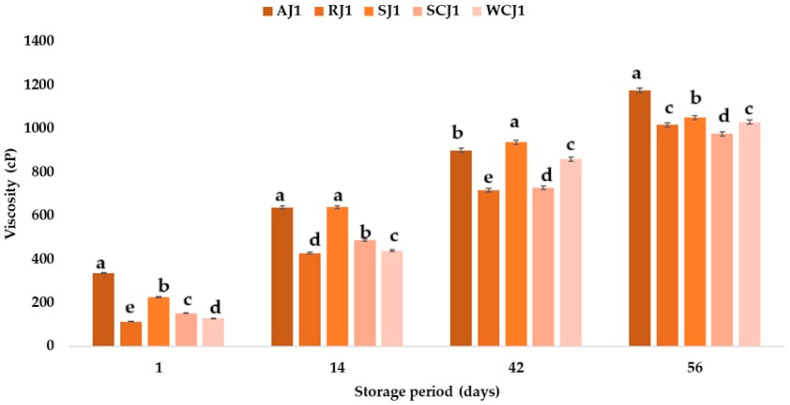
Viscosity (cP) of fruit jam samples during storage at room temperature. Means with different lowercase letters (a–e) indicate significant differences (*p* < 0.05) between samples.

**Figure 11:**
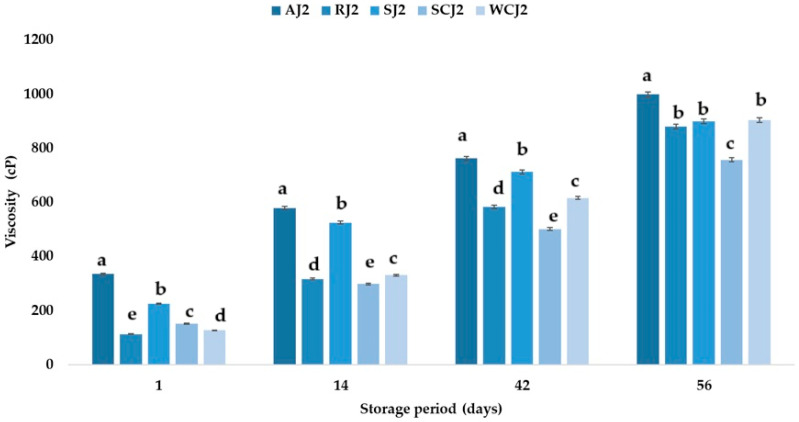
Viscosity (cP) of fruit jam samples during storage at refrigeration temperature. Means with different lowercase letters (a–e) indicate significant differences (*p* < 0.05) between samples.

The pectin content, sugar addition, and thermal processing influence jam viscosity [66]. The viscosity of the jam samples immediately after opening the jars was 112 ± 0.4 cP for the raspberry jam, 127 ± 0.2 cP for the white cherry jam, 151 ± 0.4 cP for the sour cherry jam, 224 ± 0.4 cP for the strawberry jam, and 334 ± 0.8 cP for the apricot jam. These values are lower than those reported by Hassan et al. [67] for fruit jams (734.21 cP). Shoaei et al. [68] determined a jam viscosity of 507.81 cP at refrigeration temperature and 437.5 cP at room temperature. During the storage of the jam samples, an increase in viscosity was observed regardless of the storage temperature (Figure 10 and Figure 11); however, the values obtained for the jams stored at refrigeration temperature were lower than those obtained for the jams stored at room temperature.

An increase in viscosity during jam storage was also observed by Shoaei et al. [68], but they reported higher values for jams stored at refrigeration temperature. After 56 days of storage, the highest viscosity values were determined for the apricot jam samples, at 998 ± 0.7 cP (at refrigeration temperature) and 1174 ± 0.6 cP (at room temperature), respectively, while the lowest were for the sour cherry jam samples, at757 ± 0.2 cP (at refrigeration temperature) and 975 ± 0.2 cP (at room temperature). At the end of the storage period, the raspberry and strawberry jam samples had viscosity values below 900 cP (samples stored at refrigeration temperature) and slightly above 1000 cP (samples stored at room temperature), and for the white cherry jam samples, values of 903 ± 0.8 cP and 1029 ± 0.1 cP were determined. The results show that, for the viscosity, an increase in this parameter was recorded in all the samples; this was mainly due to the amount of pectin in each sample, but also to the fact that the samples presented with saccharification. The pectin concentration and temperature are two factors which influence the viscosity of a food product. The viscosity of pectin solutions is increased by sucrose, which means that sugar influences the viscosity of jams, which increases with an increasing sugar concentration. An increase in the viscosity was observed across all the samples, with more pronounced thickening in those stored at room temperature (20 °C), suggesting a strong temperature dependence. This behavior may be attributable to water loss or polysaccharide interactions (e.g., pectin gelation or aggregation) that intensify under warmer conditions. While increased viscosity can enhance the perceived quality in some cases, excessive thickening may compromise spreadability and consumer satisfaction.

### 3.4. Color Changes During Storage of Fruit Jam Samples

Table 2 presents the color parameters of the jam samples during storage at room temperature (1) and at refrigeration temperature (2). None of the jam samples exceed 50 for the parameter L*, which means that the samples are dark, according to Prisacaru et al. [69]. The results show that immediately after opening the jars, the white cherry jam sample was the darkest (L* = 16.47), while the apricot jam sample was the lightest (L* = 33.21 ± 0.28). Vukoja et al. [70] reported L* values between 25.38 and 25.71 for cherry jam, while Koca and Ustun [71] determined a value of 15.09 for L* for cherry jam. Kaur et al. [72] obtained an L* value of 25.95 for strawberry jam. During storage, color changes occurred, and after 56 days of storage, it was observed that the strawberry and sour cherry jam samples were darker at room temperature and lighter at refrigeration temperature, while the apricot, raspberry, and white cherry jam samples were lighter at room temperature and darker at refrigeration temperature. Touati et al. [2] reported a reduction in apricot jam lightness during storage, which changed from an initial yellow to reddish tone, probably due to the formation of brown pigments by a Maillard reaction. Patras et al. [73] observed that the temperature influenced the lightness of strawberry jam samples during a 28-day storage period (the samples had much lower L* values at 15 °C compared to 4 °C). The strawberry, apricot, raspberry, and white cherries jams had positive values for the parameter a*, which means a red color, while the sour cherries jam had negative values for a* (green color).

In the case of the sour cherry jam, negative values of a* were obtained when the jam was stored at refrigeration temperature, while positive values were registered when this jam was stored at room temperature. The redness of the jam samples varied significantly during storage, but after 56 days of storage, the a* values of the strawberry jams were higher than the initial values, regardless of the storage temperature. For the apricot jam, the increase was higher for the sample stored at refrigeration temperature, while for the raspberry and white cherry jams, the a* values decreased after 56 days of storage at 4 °C compared to the initial values. All the jam samples had positive values for the color parameter b* (yellow); the highest value was obtained for the apricot jam (17.89), while the lowest was determined for the sour cherry jam. After 56 days of storage, the intensity of the yellow hue of the jam samples increased more for the strawberry and apricot jam samples stored at refrigerated temperatures, while for the sour cherry, raspberry, and white cherry jam samples, it increased more for the samples stored at room temperature. At the end of the storage period, the color difference was higher for the strawberry, sour cherry, apricot, and white cherry jam samples stored at refrigerated temperatures, compared to those stored at room temperature. The jams lost their specific colors under the latter storage conditions [70].

Furthermore, the progressive color degradation in all the jam samples during storage—regardless of temperature—highlights the susceptibility of fruit pigments (e.g., anthocyanins and carotenoids) to oxidation and degradation. Since visual appeal is a primary determinant of consumer acceptance, such color loss further emphasizes the necessity of proper storage conditions to maintain product quality.

### 3.5. Total Number of Yeasts and Molds

Dilutions were performed for the determination of the total number of yeasts and molds, both in the samples stored at ambient temperature and in those stored under refrigeration conditions, and cultivated on a specific culture medium. The apricot and sour cherry jam samples did not show any yeasts or molds immediately after opening the jars, the strawberry and raspberry jam samples showed an approximately equal number of yeasts and molds, and the white cherry jam sample showed the highest number of yeasts and molds among all the samples investigated (Table 3).

The results obtained show an increasing evolution in the number of micro-organisms during storage, both in the case of the samples stored at ambient temperature and those stored under refrigeration conditions (Table 3). As expected, the growth of yeasts and molds was higher in the jam samples stored at room temperature. After 14 days of storage, it was observed that the highest number of yeasts and molds was in the sour cherry jam sample (5.86 log_10_ CFU/g) stored at room temperature and the strawberry sample (4.56 log_10_ CFU/g) stored at refrigeration temperature. At the end of the storage period at room temperature, the total number of yeasts and molds was between 6.43 and 6.79 log_10_ CFU/g for the strawberry and sour cherry jams and apricot jam, respectively, while in the case of the samples stored at refrigeration temperatures, the total number of yeasts and molds was between 5.95 and 6.43 log_10_ CFU/g.

### 3.6. Total Number of Aerobic Mesophilic Germs (TAMG)

The determination of the total number of aerobic germs was carried out by dilutions of both the samples stored at ambient temperature and those stored under refrigeration conditions and cultivation on a specific culture medium. The results obtained show an increasing evolution in the number of bacteria during storage, both in the case of the samples stored at ambient temperature and in those stored under refrigeration conditions (Table 4).

The results show that after 14 days of storage at room temperature, the TAMG increased the most, by 63%, in the white cherry jam sample, and in the other remaining samples it increased between 34 and 39%. In the case of the samples stored at refrigeration temperature, after 14 days it was observed that the increase in the TAMG was much lower, between 9% (strawberry jam) and 34% (white cherry jam). The increase in the TAMG was lower between day 14 and day 42, between 5% and 23% (for the cherry and apricot jam samples stored at room temperature, respectively), whereas the increase was higher for the samples stored at refrigeration temperature, between 23 and 39%.

The microbiological monitoring revealed a dynamic pattern of microbial growth over time. Notably, the initial absence of yeasts and molds in the apricot and sour cherry jams may reflect an effective initial pasteurization or the presence of natural antimicrobial compounds (e.g., phenolics or organic acids). In contrast, the white cherry jam exhibited the highest initial fungal load (4.25 log_10_ CFU/g), suggesting a formulation or processing susceptibility to microbial contamination. Both the TMAG and TYM increased during storage, underlining the potential for spoilage and health risks if storage recommendations are not followed. These findings stress the importance of microbial surveillance in low-sugar jam products, particularly after opening, and support the development of stricter post-opening storage guidelines to minimize spoilage and ensure consumer safety.

### 3.7. Principal Component Analysis

**Figure 12:**
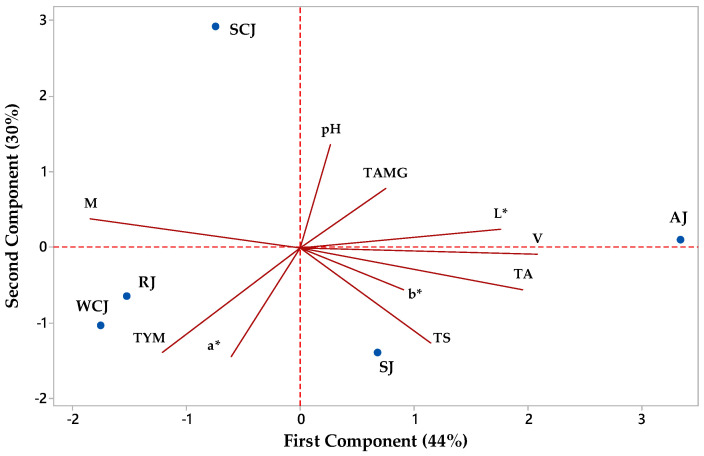
Principal component analysis biplot depicting the relationship between parameters of jam samples (AJ—apricot jam; RJ—raspberry jam; SJ—strawberry jam; SCJ—sour cherry jam; WCJ—white cherry jam; M—moisture; TA—titratable acidity; TS—total sugar; V—viscosity; L*, a*, and b*—color parameters; TYM—total number of yeasts and molds; TAMG—total number of aerobic mesophilic germs).

Figure 12 illustrates the relationship between the physicochemical and microbiological parameters of the fruit jam samples. Principal component 1 (PC1) accounted for 44% of the total variation, while PC2 accounted for 30% of the total variation. All the investigated parameters had positive loadings on PC1, except for moisture (−0.420), a* (−0.137), and TYM (−0.276), while all the investigated parameters had negative loadings on PC2, except for pH (0.458), moisture (0.128), L* (0.081), and TAMG (0.262). The apricot jam sample had positive values for both PC1 and PC2, while the raspberry and white cherry jam samples had negative values for both PC1 and PC2. The strawberry jam had positive values for PC1 and negative values for PC2, while the sour cherry jam sample had negative values for PC1 and positive values for PC2.

A Pearson’s correlation was used to investigate the obtained data, and the results show negative correlations between the pH and TYM (−0.768), pH and total sugars (−0.737), pH and a* (−0.568), titratable acidity and moisture (−0.954), total sugars and moisture (−0.684), moisture and viscosity (−0.913), moisture and L* (−0.494), viscosity and TYM (−0.538), L* and TYM (−0.644), a* and TAMG (−0.572), b* and TAMG (−0.497), and TYM and TAMG (−0.508), and higher positive correlations between the titratable acidity and viscosity (0.959), titratable acidity and total sugars (0.729), viscosity and L* (0.794), and a* and TYM (0.766).

## 4. Conclusions

Fruit jam is susceptible to spoilage due to microbial, chemical, and physical activity. This study’s results provide information on the physicochemical and microbiological stability of commercial apricot, sour cherry, white cherry, raspberry, and strawberry jams. Storage at refrigeration temperature induced changes in the physicochemical and microbiological parameters of the fruit jams, but not as much as storage at room temperature. The time–temperature interaction factor had a significant effect on the pH, titratable acidity, moisture, total sugars, viscosity, and color of the fruit jams. The number of yeasts, molds, and aerobic mesophilic germs increased during storage, as expected. The changes in the parameters were dependent upon the temperature and storage duration. Also, the type, composition, and quality of the fruit, as well as the production parameters, could have influenced the impact of jam storage on the physicochemical and microbiological stability.

Thus, the results of the present study could be used to support both producers and consumers. Consumers should consider jam producers’ recommendations on how to store jam after opening. Therefore, after opening, jams should be kept at a low temperature and consumed as soon as possible (within to 14 days), even if some producers mention a longer storage time (up to 30 days). The results of this study show that, during storage, the quality of jams begins to decline, which could have a negative impact on the health of consumers. Future studies should include sensory analyses, texture analyses, and the determination of other physicochemical parameters, and also establish the optimal storage duration for opened fruit jams through a statistical analysis of the results obtained.

## Figures and Tables

**Table 1:** Nutritional and compositional properties of jams.

Fruit Jams	Producer Specifications	Nutritional Values Declared by the Producers
Apricot jam	Ingredients: apricots, sugar, glucose syrup; acidifier: citric acid; gelling agent: pectin. Product prepared with 65 g of fruit per 100 g of finished product.	Energy value: 274 kcal/100 g Carbohydrates: 67 g/100 g Fiber: 1.4 g/100 g Protein: 0.5 g/100 g
Strawberry jam	Ingredients: strawberries (65%), sugar, glucose–fructose syrup, gelling agent (pectin), acidifier (citric acid).	Energy value: 263 kcal/100 g Fat: 0.1 g/100 g Carbohydrates: 65 g/100 g Fiber: 0.1 g/100 g Sodium: 0 g/100 g Protein: 0.5 g/100 g
Raspberry jam	Ingredients: raspberries, sugar, glucose syrup; acidifier: citric acid; gelling agent: pectin. Product prepared with 65 g of fruit per 100 g of finished product.	Energy value: 263 kCal/100 g Fat: 0 g/100 g Carbohydrates: 63.3 g/100 g Protein: 1.5 g/100 g
Sour cherry jam	Ingredients: sour cherries, glucose–fructose syrup, sugar; gelling agent: pectin; acidifier: citric acid.	Energy value: 264 kCal/100 g Fat: 0.4 g/100 g Carbohydrates: 65 g/100 g Protein: 0.8 g/100 g Salt: 0 g/100 g Fiber: 0.07 g/100 g
White cherry jam	Ingredients: white cherries, sugar; acidifier: citric acid. Product prepared with 45 g of fruit per 100 g of finished product.	Energy value: 299 kCal/100 g Fat: 0.1 g/100 g Carbohydrates: 73 g/100 g Protein: 0.5 g/100 g Salt: 0 g/100 g Fiber: 0 g/100 g

**Table 2:** Color parameters of fruit jam samples at room (1) and refrigeration (2) temperature.

Sample Name	Sample	L*	a*	b*	ΔE
Strawberry jam (1st day)	SJ	20.06 ± 0.50 ^f^	3.49 ± 0.31 ^d^	4.11 ± 0.10 ^e^	-
Strawberry jam (14th day)	SJ1	20.36 ± 0.39 ^ef^	10.39 ± 0.43 ^a^	7.74 ± 0.29 ^b^	7.82 ± 0.24 ^c^
	SJ2	21.44 ± 0.20 ^e^	6.04 ± 0.08 ^c^	5.27 ± 0.03 ^d^	3.12 ± 0.13 ^e^
Strawberry jam (42th day)	SJ1	20.74 ± 0.41 ^c^	1.20 ± 0.20 ^e^	3.39 ± 0.10 ^f^	5.26 ± 0.46 ^d^
	SJ2	29.44 ± 0.42 ^a^	3.89 ± 0.13 ^d^	5.94 ± 0.23 ^cd^	9.56 ± 0.46 ^b^
Strawberry jam (56th day)	SJ1	20.70 ± 0.43 ^d^	7.90 ± 0.64 ^b^	6.25 ± 0.37 ^c^	5.59 ± 0.61 ^d^
	SJ2	27.82 ± 0.82 ^b^	8.14 ± 0.42 ^b^	10.24 ± 0.38 ^a^	10.94 ± 0.42 ^a^
Sour cherry jam (1st day)	SCJ	21.10 ± 0.48 ^abc^	−0.11 ± 0.40 ^a^	2.63 ± 0.11 ^bc^	-
Sour cherry jam (14th day)	SCJ1	21.52 ± 1.21 ^abc^	0.28 ± 0.24 ^a^	2.66 ± 0.08 ^abc^	1.51 ± 0.35 ^a^
	SCJ2	17.80 ± 3.14 ^c^	−0.26 ± 0.14 ^a^	3.10 ± 0.23 ^ab^	3.37 ± 3.58 ^a^
Sour cherry jam (42th day)	SCJ1	20.17 ± 1.55 ^abc^	0.28 ± 0.14 ^a^	3.15 ± 0.05 ^a^	1.88 ± 0.51 ^a^
	SCJ2	25.21 ± 0.28 ^a^	−0.14 ± 0.08 ^a^	2.52 ± 0.01 ^c^	4.13 ± 0.20 ^a^
Sour cherry jam (56th day)	SCJ1	20.03 ± 2.88 ^bc^	0.43 ± 0.66 ^a^	2.88 ± 0.39 ^abc^	1.87 ± 2.03 ^a^
	SCJ2	23.66 ± 1.37 ^ab^	−0.28 ± 0.07 ^a^	2.50 ± 0.03 ^c^	2.61 ± 1.83 ^a^
Apricot jam (1st day)	AJ	33.21 ± 0.28 ^bc^	3.58 ± 0.24 ^cd^	17.89 ± 0.90 ^cd^	-
Apricot jam (14th day)	AJ1	32.55 ± 0.61 ^c^	4.31 ± 0.20 ^ab^	14.66 ± 0.44 ^e^	3.49 ± 0.42 ^ab^
	AJ2	35.97 ± 0.51 ^a^	3.39 ± 0.08 ^d^	19.25 ± 0.08 ^bc^	3.15 ± 0.60 ^ab^
Apricot jam (42th day)	AJ1	35.69 ± 1.01 ^a^	4.47 ± 0.06 ^a^	19.64 ± 0.75 ^b^	3.32 ± 0.67 ^ab^
	AJ2	36.23 ± 0.18 ^a^	2.86 ± 0.03 ^e^	17.52 ± 0.20 ^d^	3.19 ± 0.56 ^ab^
Apricot jam (56th day)	AJ1	34.94 ± 0.58 ^ab^	3.99 ± 0.19 ^bc^	17.04 ± 0.38 ^d^	2.10 ± 0.99 ^b^
	AJ2	31.55 ± 0.98 ^c^	4.67 ± 0.14 ^a^	22.72 ± 0.56 ^a^	5.28 ± 1.63 ^a^
Raspberry jam (1st day)	RJ	23.31 ± 0.16 ^f^	6.06 ± 0.05 ^a^	5.95 ± 0.03 ^d^	-
Raspberry jam (14th day)	RJ1	26.19 ± 0.35 ^bc^	5.04 ± 0.12 ^b^	5.52 ± 0.01 ^e^	3.09 ± 0.50 ^b^
	RJ2	29.36 ± 0.46 ^a^	3.59 ± 0.24 ^c^	5.86 ± 0.19 ^d^	6.54 ± 0.44 ^a^
Raspberry jam (42th day)	RJ1	24.94 ± 0.27 ^de^	6.12 ± 0.22 ^a^	6.82 ± 0.06 ^ab^	1.86 ± 0.28 ^c^
	RJ2	26.55 ± 0.26 ^b^	4.82 ± 0.13 ^b^	6.63 ± 0.07 ^bc^	3.54 ± 0.42 ^b^
Raspberry jam (56th day)	RJ1	25.62 ± 0.20 ^cd^	6.46 ± 0.06 ^a^	6.91 ± 0.03 ^a^	2.53 ± 0.33 ^bc^
	RJ2	24.59 ± 0.38 ^e^	4.83 ± 0.24 ^b^	6.49 ± 0.15 ^c^	1.88 ± 0.18 ^c^
White cherry jam (1st day)	WCJ	16.47 ± 1.19 ^e^	6.63 ± 0.11 ^a^	15.10 ± 0.56 ^b^	-
White cherry jam (14th day)	WCJ1	37.84 ± 0.32 ^a^	3.59 ± 0.12 ^c^	26.57 ± 0.57 ^a^	24.46 ± 1.28 ^a^
	WCJ2	38.76 ± 0.17 ^a^	2.61 ± 0.06 ^d^	26.03 ± 0.26 ^a^	9.64 ± 2.42 ^b^
White cherry jam (42th day)	WCJ1	25.78 ± 1.02 ^cd^	6.30 ± 0.31 ^a^	12.75 ± 0.90 ^c^	13.69 ± 1.45 ^b^
	WCJ2	27.19 ± 0.52 ^bc^	6.71 ± 0.06 ^a^	12.73 ± 0.23 ^c^	25.16 ± 1.07 ^a^
White cherry jam (56th day)	WCJ1	29.16 ± 0.32 ^b^	4.82 ± 0.39 ^b^	10.34 ± 0.11 ^d^	10.98 ± 1.82 ^b^
	WCJ2	23.65 ± 2.44 ^d^	3.62 ± 0.04 ^c^	7.54 ± 0.38 ^e^	11.03 ± 2.77 ^b^

Values are means and standard errors of three determinations (considering one 1–56 days data group for each type of jam separately). Values with different letters within one column per each type of jam are significantly different (*p* < 0.05).

**Table 3:** Yeast and mold growth in fruit jams during storage at room and refrigeration temperatures (in log_10_ CFU/g).

Storage Day	Sample	Total Number of Yeasts and Molds (TYM)	
Day 1	AJ	0.00	
	RJ	3.95 ± 0.01 ^b^	
	SJ	3.95 ± 0.00 ^b^	
	SCJ	0.00	
	WCJ	4.25 ± 0.03 ^a^	
Day 14		Room temperature	Refrigeration temperature
	AJ	4.95 ± 0.05 ^c^	4.56 ± 0.06 ^a^
	RJ	4.95 ± 0.07 ^c^	4.26 ± 0.05 ^b^
	SJ	5.43 ± 0.05 ^b^	4.56 ± 0.06 ^a^
	SCJ	5.86 ± 0.01 ^a^	3.95 ± 0.04 ^c^
	WCJ	4.95 ± 0.05 ^c^	3.95 ± 0.04 ^c^
Day 42	AJ	5.95 ± 0.01 ^c^	5.76 ± 0.07 ^c^
	RJ	6.26 ± 0.03 ^b^	5.95 ± 0.04 ^b^
	SJ	6.43 ± 0.04 ^a^	5.95 ± 0.04 ^b^
	SCJ	5.95 ± 0.01 ^c^	6.26 ± 0.05 ^a^
	WCJ	6.43 ± 0.03 ^a^	5.78 ± 0.07 ^c^
Day 56	AJ	6.79 ± 0.02 ^a^	5.95 ± 0.04 ^b^
	RJ	6.65 ± 0.01 ^b^	5.95 ± 0.04 ^b^
	SJ	6.43 ± 0.06 ^c^	5.95 ± 0.02 ^b^
	SCJ	6.43 ± 0.05 ^c^	6.43 ± 0.01 ^a^
	WCJ	6.55 ± 0.03 ^b^	5.95 ± 0.04 ^b^

Values are means and standard errors of three determinations (considering one AJ–WCJ data group for each day separately). Values with different letters within one column per each day of determination are significantly different (*p* < 0.05).

**Table 4:** Total number of aerobic mesophilic germs growth in fruit jams during storage at room and refrigeration temperatures (in log_10_ CFU/g).

Storage Day	Sample	Total Number of Aerobic Mesophilic Germs (TAMG)	
Day 1	AJ	4.65 ± 0.03 ^ab^	
	RJ	4.73 ± 0.02 ^a^	
	SJ	4.56 ± 0.06 ^b^	
	SCJ	4.73 ± 0.01 ^a^	
	WCJ	3.95 ± 0.04 ^c^	
Day 14		Room temperature	Refrigeration temperature
	AJ	6.46 ± 0.01 ^a^	5.21 ± 0.01 ^a^
	RJ	6.45 ± 0.05 ^ab^	5.32 ± 0.05 ^a^
	SJ	6.28 ± 0.06 ^c^	4.95 ± 0.04 ^b^
	SCJ	6.35 ± 0.02 ^bc^	5.32 ± 0.06 ^a^
	WCJ	6.43 ± 0.01 ^ab^	5.30 ± 0.06 ^a^
Day 42	AJ	7.97 ± 0.06 ^a^	6.43 ± 0.01 ^d^
	RJ	7.00 ± 0.01 ^c^	6.91 ± 0.02 ^b^
	SJ	7.26 ± 0.05 ^b^	6.86 ± 0.03 ^b^
	SCJ	6.65 ± 0.03 ^d^	7.07 ± 0.01 ^a^
	WCJ	7.26 ± 0.02 ^b^	6.56 ± 0.05 ^c^
Day 56	AJ	9.20 ± 0.04 ^b^	7.65 ± 0.03 ^b^
	RJ	9.29 ± 0.07 ^b^	7.65 ± 0.02 ^b^
	SJ	9.43 ± 0.02 ^a^	9.28 ± 0.04 ^a^
	SCJ	8.82 ± 0.01 ^c^	9.31 ± 0.01 ^a^
	WCJ	8.55 ± 0.04 ^d^	9.34 ± 0.03 ^a^

Values are means and standard errors of three determinations (considering one AJ–WCJ data group for each day separately). Values with different letters within one column per each day of determination are significantly different (*p* < 0.05).

## Data Availability

The original contributions presented in this study are included in this article, further inquiries can be directed to the corresponding authors.
